# Fertilizer potential of biochar and ryegrass productivity in metal-contaminated soil

**DOI:** 10.3389/fpls.2024.1475939

**Published:** 2025-01-09

**Authors:** Joao Arthur Antonangelo, Joao Luis Bigatao Souza, Hailin Zhang

**Affiliations:** ^1^ Department of Crop and Soil Sciences, Washington State University, Pullman, WA, United States; ^2^ Department of Land Resources and Environmental Sciences, Montana State University, Bozeman, MT, United States; ^3^ Department of Plant and Soil Sciences, Oklahoma State University, Oklahoma, OK, United States

**Keywords:** lime effect, biochar application, fertilizer amendment, phosphorus and potassium, ryegrass production

## Abstract

**Introduction:**

Response to fertilization with biochar in contaminated soils for forage crops lacks comprehensive understanding. This study delves into the role of biochar in enhancing soil pH and phosphorus (P) and potassium (K) availability for ryegrass (*Lolium perenne*) in clay and silt loam metal-contaminated soils.

**Methods:**

Two pot experiments were conducted using switchgrass-derived biochar (SGB) and poultry litter-derived biochar (PLB) with varying biochar application rates: one without plants and the other with ryegrass.

**Results:**

Results demonstrated a significant rise in soil pH with increasing biochar rates, particularly notable for the PLB experiment with plants, attributed to PLB’s superior buffer capacity. PLB significantly improved ryegrass productivity, evident in germination percentage, plant population, and biomass, especially at a 0.5-1% biochar application rate. However, excessive biochar application (2-4%) hindered plant growth.

**Discussion:**

PLB at 1% application sufficed to barely surpass critical P and K thresholds for optimal ryegrass production, whereas SGB fell short of meeting these thresholds, highlighting the importance of biochar feedstock selection. While biochar shows promise for metal remediation and nutrient enhancement, caution is advised against excessive application, considering potential nutrient contamination risks based on feedstock variations.

## 1 Introduction

Biochar, a carbon-rich product derived from the thermo-chemical decomposition of biomass under limited oxygen conditions, has garnered attention for its potential to enhance soil quality. Extensive research has highlighted its efficacy in amending highly degraded and nutrient-poor soils, thereby boosting crop productivity (Basak et al., 2022; El-Naggar et al., 2019; Jeffery et al., 2017; Hussain et al., 2017). Existing studies have focused on its application in tropical soils (Basak et al., 2022) given the biochar’s potential to neutralize soil acidity, overlooking its potential benefits in temperate regions (Jeffery et al., 2017) where its liming effect could be particularly advantageous. Moreover, while biochar’s role in ameliorating metal-contaminated soils has been explored, its capacity to serve as a source of essential nutrients remains under investigated. Notably, a study by Antonangelo and Zhang (2019) demonstrated that biochar application in a neutral-to-alkaline metal-contaminated soil (Tar Creek) led to pH elevation in a temperate region, enhanced ryegrass growth, and reduced metal availability, contrary to prevailing literature. Yet, that study did not delve into biochar’s potential as a fertilizer source, a critical aspect for sustainable agriculture, where utilizing waste-based materials simultaneously as fertilizers and contaminant suppressors could offer promising solutions for land degradation. Further exploration of biochar’s multifaceted role in enhancing soil fertility and mitigating contamination is imperative for advancing sustainable agricultural practices.

The Tar Creek area, situated within the tri-state regions of Oklahoma, Kansas, and Missouri, has been designated as a Superfund site by the US Environmental Protection Agency (EPA) due to its severe metal pollution, rendering it one of the most contaminated sites globally (Neuberger et al., 2008). Given this context, employing biochar emerges as a viable strategy for the immobilization of heavy metals. Furthermore, Perennial ryegrass (*Lolium perenne*), characterized by rapid growth, high yield potential, robust resistance to toxic metals, and high adaptability to harsh environments such as tailing areas with severe metal pollution, presents itself as a promising candidate for forage cultivation (Ji et al., 2020). Despite these promising aspects, existing research have primarily focused on the combined effects of biochar on soil properties and row crop yields, thus leaving a research gap concerning the relationship between soil nutrient dynamics and the integrated impacts of biochar and forage crop management practices (Wang et al., 2020; Biederman et al., 2017). Beyond the environmental benefits associated with metal immobilization, attention must be paid to the short-term adverse effects of biochar application on soil nutrient levels in pastures (Hays et al., 2023), particularly concerning potential over-application of phosphorus (P) and its subsequent water quality concerns. Hence, determining an optimal rate of biochar application becomes imperative, striking a balance between metal immobilization and maintaining adequate nutrient levels essential for crop production.

Several studies have indicated that the utilization of biochar enhances various plant growth attributes, including germination percentage, shoot and root growth, and overall plant survival (Wang et al., 2019; Teodoro et al., 2020). As far as a soil amendment and nutrient source, manure-derived biochars are recognized for their notable liming potential and fertilizer value, offering essential nutrients such as P, potassium (K), calcium (Ca), and magnesium (Mg) to both topsoil and plants (Wang et al., 2012; Subedi et al., 2016). In recent times, there have been notable advancements in the development, synthesis, application, and comprehension of the mechanisms involved in biochar-based fertilizers (Melo and Sánchez-Monedero, 2024). These fertilizers are particularly recognized for their slow-release characteristics and improved efficiency (Wang et al., 2022), potentially leading to reduced carbon emissions in agricultural practices. Consequently, careful attention must be given to application rates to ensure they do not exceed 100% of the soil’s optimal nutrient levels for the cultivation of cash crops. In contrast, biochars derived from wood and green waste materials are reported to contain lower mineral constituents but exhibit a higher carbon content compared to manure-derived biochars (Hassan et al., 2020).

The urgency of sustainable land management and restoration to support agricultural practices that can nourish the expanding population is increasingly evident. On this basis, the evaluation of biochar’s fertilizing efficacy in contaminated soil for forage production becomes pivotal, as it encompasses its multifaceted role in enhancing soil health, nutrient provision, metal immobilization, crop yield augmentation, and environmental mitigation. Such assessment is fundamental for establishing sustainable soil management strategies and securing economic feasibility. Notably, identifying biochar application rates capable of simultaneously immobilizing contaminants and optimizing nutrient availability, without the risk of overapplication, underscores a mutualistic outcome, thereby promoting both agricultural productivity and environmental stewardship.

In this context, this study aims to fill a notable gap in understanding the impacts of biochar fertilization on forage crop productivity in metal-contaminated soils, with a specific emphasis on three key aspects: (i) enhancement of soil pH through a comprehensive assessment of biochar pH-buffering capacity, (ii) improvement of P and K availability to ryegrass, and (iii) evaluation of the ideal application rate to minimize metal mobility while ensuring sufficient levels of P and K without overapplication so P is not excessively built up. To our knowledge, it is a novel concept to suggest that biochars can simultaneously function as metal immobilizers and fertilizers. We hypothesize that the optimal rate of biochar for effective metal immobilization, due to its enhanced carbon stability, might also supply essential nutrients for ryegrass growth. To achieve this, two pot experiments were conducted employing biochars derived from distinct feedstocks: one focused on soil without plants, while the other incorporated ryegrass to examine the effects of varying biochar application rates on soil pH, sufficiency levels of P and K, and ryegrass responses. It is important to note that this study does not delve into the dynamics of heavy metals, as this aspect has been previously investigated by Antonangelo and Zhang (2019), which is appropriately referenced for further context.

## 2 Materials and methods

### 2.1 Biochar Production

The feedstocks (raw material) used to produce biochars were presented in Antonangelo et al. (2019). Switchgrass and Poultry litter-derived biochar (SGB and PLB, respectively) were generated through slow pyrolysis at 350 and 700°C. Prepared samples (0.5–1.5 kg) underwent pyrolysis in a Lindburg electric box furnace with a gas-tight retort (Model 51662; Lindburg/MPH, Riverside, MI), following the protocol by Cantrell et al. (2012). Pyrolysis conditions included a 1-hour equilibration hold at 200°C under industrial-grade N_2_ flow (15 L min^−1^), temperature ramping to the desired level (2.52°C min^−1^ for 350°C; 8.33°C min^−1^ for 700°C), a 2-hour hold at maximum temperature under N_2_ flow (1 L min^−1^), and cooling to 100°C (4.25°C min^−1^). Biochars were then cooled to room temperature in an inert N_2_ atmosphere, following the method detailed by Cantrell et al. (2012). Ground coarse biochar materials passed through 1- and 0.25-mm mesh screens for, respectively, biochar analyses and subsequent experiments. A summary of biochar analyses pertinent for this study is included in **Table 1**. Detailed description of methods and additional analyses can be found in Antonangelo et al. (2019).

**Table 1:** Physicochemical properties of biochars derived from two feedstocks and from the Tar Creek soil.

Parameter	Method	Unit	SGB350	PLB350	SGB700	PLB700	Soil
pH	Water	-	5.2	7.4	10.1	10.2	6.1
TN	Dry combustion	%	0.9	4.1	0.7	1.6	0.17
TC	Dry combustion	%	42.6	38.4	31.4	27.8	2.1
Ash	Loss on ignition	%	2.7	29.8	4.4	45.9	-
P	EPA 3050B	%	0.1	3.0	0.2	4.0	0.03
K	EPA 3050B	%	0.3	6.0	0.4	8.0	0.09
Ca	EPA 3050B	%	0.5	4.0	0.8	5.0	0.39
Mg	EPA 3050B	%	0.3	1.0	0.3	2.0	0.12
S	EPA 3050B	%	0.07	1.0	0.04	1.0	0.08
Fe	EPA 3050B	mg kg^-1^	95	3313	111	6903	13029
Zn	EPA 3050B	mg kg^-1^	39	1018	54	1477	1765
Cu	EPA 3050B	mg kg^-1^	17	209	23	253	12.8
B	EPA 3050B	mg kg^-1^	<LOD	104	<LOD	103	242.4
P	Mehlich-3	mg kg^-1^	120	6293	254	8425	25
K	Mehlich-3	mg kg^-1^	193	24498	616	54504	118
Ca	Mehlich-3	mg kg^-1^	323	4862	1575	5690	2094
Mg	Mehlich-3	mg kg^-1^	202	3616	541	6869	154
Fe	DTPA-sorbitol	mg kg^-1^	1.0	2.7	2.7	122.4	35.3
Zn	DTPA-sorbitol	mg kg^-1^	1.0	22.8	3.8	29.8	221
Cu	DTPA-sorbitol	mg kg^-1^	0.6	10.7	1.2	9.8	1.77
B	DTPA-sorbitol	mg kg^-1^	<LOD	8.4	<LOD	5.6	0.12

TC, total carbon; TN, total nitrogen; SGB, switchgrass-derived biochar; PLB, poultry litter-derived biochar; LOD, limit of detection. “- “: non-applicable. This table is an adaptation of the data presented in the work of Antonangelo and Zhang (2019).

### 2.2 Biochar pH and buffering capacity

The pH of each biochar was determined in DI water at a 1:5 (w/w) ratio following methods outlined by Yuan et al. (2011). Samples were mixed with DI water in 50 mL centrifuge tubes, shaken at 220 rpm for 1 hour, and pH was measured using an Orion Star A221 pH electrode (Thermo Scientific). Results can be found in **Table 1**.

To assess the proton neutralization capacity, acid-base titration was conducted as described by Yuan et al. (2011). Half a gram (0.5 g) of biochar samples was placed in 125 mL serum bottles, followed by the addition of 20 mL of DI water (1:40 biochar to solution ratio). Each bottle was stirred on a magnetic stirrer for 2 hours at 25°C. Subsequently, samples were titrated with 0.1 M HCl at 25°C until reaching the endpoint at pH 2.0 using an automatic titrator (TIM840 Titration Manager, Hach Company, Loveland, CO, USA) with continuous stirring. The titration rate was maintained at 0.5 mL min^-1^, with data collected every 6 seconds.

Based on the prior research conducted by Antonangelo et al. (2019), the biochars derived from pyrolyzing poultry litter (PLB) and switchgrass (SGB) at 700°C (referred to as SGB700 and PLB700 for pH-buffering analysis) were identified as better candidates for metal remediation when compared to those produced at 350°C from the same feedstocks (designated as SGB350 and PLB350) thus were also used to evaluate the fertilizer potential of biochars in this current study. Additionally, it is noteworthy to mention that, also due to limited material availability, subsequent potting experiment where ryegrass was cultivated, was not feasible for biochars pyrolyzed at 350°C. However, for the present investigation, the pH and buffering capacity of all biochars was evaluated to facilitate comparative analyses, thereby elucidating the potential lime effect exerted by biochars produced from distinct feedstocks and across varying pyrolysis temperatures. Finally, those pyrolyzed at 700°C demonstrated superior pH improvement. Consequently, using biochar pyrolyzed at 700°C was more appropriate for ryegrass growth.

### 2.3 Soil

We selected the soil from a contaminated site for our experiment, forming a composite sample from three subsamples to minimize variability. These subsamples, collected from depths of 0-15 cm, originated from locations near chat piles (debris from lead-zinc milling operations) formerly used for agriculture, and back yards within the Tar Creek region of Picher, Ottawa County, Oklahoma, known for significant metal contamination. Despite this, various revitalization efforts have transformed the area for residential, commercial, public, and agricultural uses, with ongoing developments for recreational, cultural preservation, and agricultural purposes. Initial soil chemical attributes are detailed in Antonangelo and Zhang (2019), while pertinent soil analyses for the present work are presented in **Table 1**. The soil texture was obtained using the hydrometer method in processed samples, which measures particle size distribution. A dried and ground soil sample was mixed with a dispersing agent (0.1 M sodium hexametaphosphate) and water, then placed in a graduated cylinder. A hydrometer was used to measure the suspension’s density at intervals to reflect the settling rates of sand, silt, and clay. These readings were used to calculate particle percentages and classify the soil texture as clay loam, which tends to increase metal sorption due to the high surface area.

### 2.4 Experiments

Experiment 1 involved plastic containers filled with 200 g of 2 mm sieved soil. Five rates of 0.25-mm sieved SGB and PLB (0%, 1%, 2%, 4%, and 8% w/w) were added to the soils, followed by moistening to 75% field capacity with RO (reverse osmosis) water. Incubation lasted 70 days at 25°C with weekly weight checks to maintain moisture levels. Gas exchange was facilitated by keeping samples open. This trial followed a complete randomized design (CRD) with 3 replications.

Experiment 2 utilized plastic pots filled with 1.2 kg of dried, sieved soil and applied a lower range of SGB and PLB rates (0%, 0.5%, 1%, 2%, and 4% w/w) suitable for ryegrass growth. Incubation at 75% field capacity lasted 30 days before sowing ryegrass at a rate of 30 kg ha^−1^. Each pot received an identical amount of nitrogen (N) based on soil tests for grass production, with the N provided by biochars being subtracted from the total amount. This approach ensured that any difference observed in the treatments was solely due to the application of biochar. Additionally, we controlled variations in phosphorus (P), potassium (K), and micronutrient levels to ensure that the results were primarily influenced by these factors. Ryegrass growth was monitored for 75 days in a controlled growth chamber with humidity maintained between 75% and 95%. The temperature alternated between 20°C for the initial two weeks and 14°C with 14 hours of simulated daylight and 10°C with 10 hours of simulated darkness thereafter. This experiment also followed a CRD with 3 replications, implementing weekly plot rotations to minimize chamber effects.

### 2.5 Soil analysis

At the end of both experiments, soil samples underwent initial drying and sieving to 2 mm before analysis. Soil pH was measured in deionized water using a 1:1 soil-to-water ratio (Thomas, 1996). Soil organic carbon (OC) was analyzed via dry combustion employing a LECO Truspec C/N analyzer (St. Joseph, MI). Plant-available P, K, Ca, and Mg were extracted using Mehlich 3 solution (Mehlich, 1984), while sulfur (S) was extracted by shaking 10 g of soil in 25 mL of 0.008 M MCP (monocalcium phosphate) for 30 min and the suspension was then filtered (Carter, 1993). Extraction of available micronutrients iron (Fe), copper (Cu), and boron (B) involved adding 20 mL of DTPA-sorbitol solution to 10 g of soil and shaking for 2 hours, with a modification for simultaneous extraction and determination of B, Cu, and Fe via the addition of 0.2 M sorbitol. All extracts were quantified using inductively coupled plasma atomic emission spectroscopy (ICP-AES).

Zinc (Zn) was excluded from analysis due to its potentially toxic levels, classified as a contaminant (Antonangelo and Zhang, 2019), with a total concentration of 1765 mg kg^-^¹ and bioavailable fraction at 221 mg kg^-^¹ under the initial experimental conditions (**Table 1**). At a soil pH of 6.1, Zn remains relatively soluble and available for plant uptake, as Zn solubility decreases sharply in more alkaline conditions. In this pH range, plants and microbes can readily absorb Zn, increasing the risk of toxicity, especially at high bioavailable levels like 221 mg kg^-^¹. This bioavailability could be influenced by P availability, as higher P levels can sometimes lead to Zn precipitation, reducing its mobility. However, with P levels at only 25 mg kg^-^¹ (**Table 1**), Zn remains largely unbound and bioavailable, contributing to its high availability and potential for toxicity at these initial conditions.

### 2.6 Plant analyses

In Experiment 2, germinated seeds were tallied to determine the germination rate. Additionally, the number of stands per pot was manually counted, before plant harvesting, to represent the plant population as number (#) of plants per pot. Following harvest, shoots and roots underwent separation, washing with deionized water, and oven-drying at 105°C until reaching constant weight, with subsequent recording of their weights, then plant materials were mechanically ground for further analysis.

For nutrient concentration analysis, ground plant materials underwent nitric acid digestion. Specifically, 0.5 g of ground plant materials were predigested with 10 ml of trace metal grade HNO_3_ for 1 hour in the HotBlockTM Environmental Express block digester. Subsequently, the digestion products were heated to 115°C for 2 hours and diluted with deionized water to a final volume of 50 mL, following the method described by Westerman (1990). The resulting digested samples were analyzed for P, K, Ca, Mg, S, Fe, Cu, and B using ICP-AES. Nitrogen (N) concentration was assessed in dried and ground tissues via dry combustion and determined via LECO, as previously described for soil OC.

### 2.7 Data analysis

The study employed a nonlinear regression analysis utilizing the NLIN procedure of SAS version 9.4 to explore the relationship between hydrogen ion (H^+^) addition and biochar pH change, employing both linear- and quadratic-with-lower-plateau models. Models were deemed significant at the p<0.05 level post meeting convergence criteria. Boxplot analyses were conducted using JMP Pro 17 to visualize the entire dataset of measurements, encompassing all replicate data for soil pH. Subsequently, a two-way ANOVA was applied to assess the response variables, with treatment averages (comprising biochar rates and feedstock types) compared using the Tukey test at α = 0.05. Additionally, regression analyses and Pearson correlation were executed in JMP Pro 17 on select variables across the entire dataset at α = 0.05. Graphs and tables were generated using Excel.

We investigated the soil P and K increments with increasing biochar application to assess sufficiency levels since N applications are determined based on yield goals (for comprehensive evaluation of N dynamics, refer to Antonangelo and Zhang, 2021). Additionally, P and K levels in biochars used in both experiments were higher for PLB (**Table 1**), serving as comparison benchmarks among all biochars. Thus, it can be inferred that, if our biochar is intended as a fertilizer source, achieving desired levels of P and K in the soil is recommended, as overapplication of both nutrients would occur if biochar were solely relied upon as an N source.

Stepwise Multiple Linear Regression (SMLR) analysis was also conducted. For this, a comprehensive set of soil chemical attributes, encompassing organic carbon (OC), pH, macro- (P, K, Ca, Mg, and S), and micronutrients (Cu, Fe, and B), was employed as independent variables to forecast the ryegrass growth parameters under investigation. Notably, Zn was excluded from consideration due to its status as a contaminant. The SMLR model was constructed utilizing the entire dataset of measurements within JMP Pro 15, employing the backward elimination method. Initially, the model incorporated all variables, after which the least statistically significant ones were systematically removed. The resultant variables constituted the final SLR model. In this investigation, variable elimination was guided by the Akaike Information Criterion corrected for small sample sizes (AICc), recognized for its suitability in identifying optimal models for predictive purposes.

## 3 Results and discussion

### 3.1 Biochar buffering capacity

**Figure 1:**
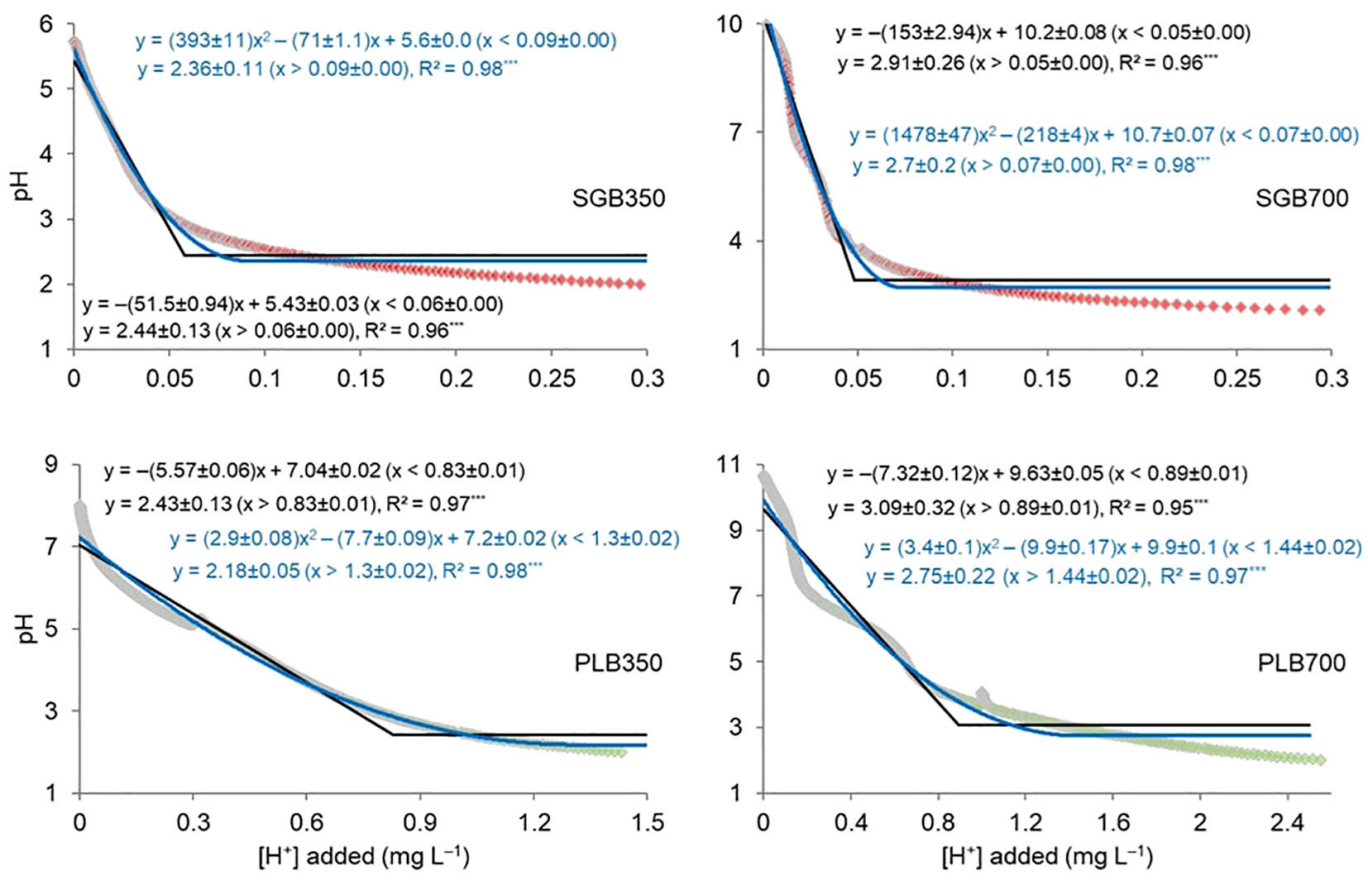
Linear- and quadratic-with lower plateau relationship for adding hydrogen ions (H^+^) and biochar pH change. The “±” symbol indicates the standard deviation of non-linear regression coefficients. ***: Models are significant at p<.0001. SGB: switchgrass-derived biochar. PLB: poultry litter-derived biochar. The joint point (*njoint*) from both models clearly indicates the buffering capacity of biochar is more affected by the feedstock than the pyrolysis temperature regardless of the biochar’s initial pH. Red and green data points comprise the titration curves of SGB and PLB, respectively.

The findings from the analysis of the relationship between H^+^ addition and biochar pH change revealed important insights into the buffering capacity of biochar, influenced notably by its feedstock source. The observed linear and quadratic relationships, coupled with lower plateaus (**Figure 1**), underscore the complex interplay between H^+^ and biochar pH, with significant implications for soil amendment strategies and environmental remediation efforts.

The joint point (*njoint*) derived from both linear- and quadratic-plateau models served as a critical indicator of the buffering capacity of biochar, shedding light on its response to changes in H^+^ concentrations. Notably, our results show that there is no difference of joint points when comparing biochars derived from the same feedstock pyrolyzed at different temperatures, regardless of the fitted model (**Figure 1**). This highlights that the buffering capacity of biochar is more profoundly impacted by the feedstock source rather than the pyrolysis temperature, irrespective of the biochar’s initial pH. The limited influence of pyrolysis temperature on the buffering capacity of biochar is consistent with previous studies indicating that feedstock characteristics exert a more dominant influence on biochar properties than processing conditions (Woolf et al., 2010; Liang et al., 2006). This highlights the potential for tailored biochar production processes focusing on feedstock diversity and optimization rather than exclusively manipulating pyrolysis parameters. Across various models and pyrolysis temperatures, the requisite amount of H^+^ necessary to adjust the initial pH of biochar to approximately 2.0 varies between 0.05 and 0.09 mg H^+^ L^-1^ for SGB and between 0.83 and 1.44 mg H^+^ L^-1^ for PLB (**Figure 1**).

Therefore, the greater buffering capacity was exhibited by PLB, which is about sixteen (~16) folds of that observed with SGB (**Figure 1**), which underscores the significance of feedstock composition in dictating biochar’s efficacy as a soil amendment agent. This observation aligns with previous studies highlighting the influence of feedstock characteristics on biochar properties, including pH, surface area, and chemical composition (Kalu et al., 2022; Liu et al., 2013). The enhanced buffering capacity of PLB can be attributed to its nitrogen-rich compounds derived from animal manure, which contribute to increased cation exchange capacity (CEC) and buffering efficiency (Lehmann and Joseph, 2024; Laird, 2008). In contrast, SGB, derived from green waste material, may exhibit comparatively lower buffering capacity due to differences in organic composition, given the more prominent soil OC enhancement when this biochar is applied in comparison to PLB (**Supplementary Tables S1**, **S2**), and nutrient content. These findings once more emphasize the importance of considering feedstock selection in biochar production to optimize its functional properties for specific applications, such as soil pH management and nutrient retention.

### 3.2 Soil pH

**Figure 2:**
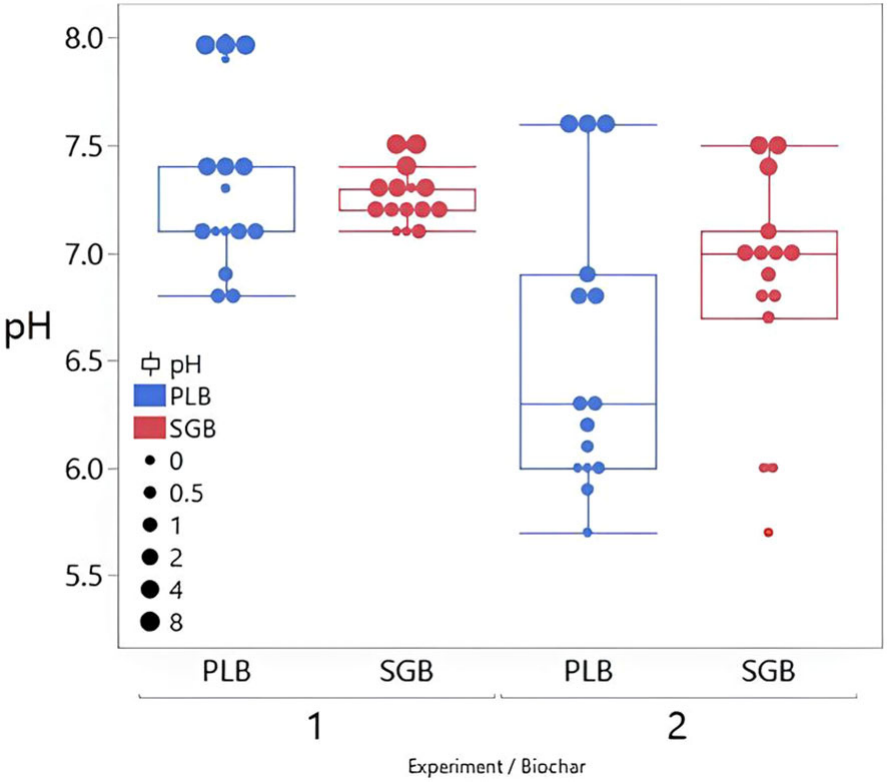
Effect of Biochar Application Rates on Soil pH: Comparative Analysis at 10 and 15 Weeks of Incubation for Two Experimental Trials. SGB: Biochar Derived from Switchgrass. PLB: Biochar Derived from Poultry Litter. The boxes illustrate the interquartile range (25^th^ to 75^th^ percentile), whiskers represent 1.5 times the interquartile range, median values are denoted by horizontal lines, and outliers are represented by individual data points outside the whiskers. Each box plot has 15 observations (*n*=15).

There is a clear trend of increasing soil pH with increased biochar application rates (**Figure 2**; **Supplementary Tables S1**, **S2**). Notably, the effect is more pronounced for PLB compared to SGB, particularly evident in the experiment with plants. This heightened response is attributed to the superior buffer capacity of PLB (**Figure 1**), indicating its effectiveness in raising soil pH levels over time (**Figure 2**).

The observed increase in soil pH with escalating PLB application rates underscores the potential of this biochar as a soil amendment for pH modification. This phenomenon aligns with previous studies indicating biochar’s ability to alter soil pH by influencing various chemical processes, including CEC and base saturation (Bolan et al., 2023). Especially in the case of PLB, there is a clear correlation between the increase in biochar application rates and the concurrent rise in soil CEC and pH (**Supplementary Tables S1**-**S4**).

Our findings challenge the conclusions of Jeffery et al. (2017), who suggested that temperate soils with neutral to slightly alkaline pH levels exhibit minimal responsiveness to biochar supplementation in terms of pH elevation. While our first experiment did show a modest increase in soil pH, rising from 6.8 to 8.0 across different biochars, which aligns with Jeffrey et al.’s observations of a systematic decline in pH responsiveness as initial pH increases, our subsequent experiment with initially acidic soils revealed a more pronounced alkalizing effect of biochar. In this case, pH levels increased from 5.7 to 7.6 across various biochars, consistent with the findings of El-Naggar et al. (2019); Hussain et al. (2017), and Williams et al. (2023). The observed differences in pH changes across experiments cannot be solely attributed to variations in biochar application rates. It is crucial to note that the absence of plants in the initial experiment resulted in the lack of root exudates, nutrient uptake, and possibly reduced microbial activity, which could lead to different pH responses compared to soils with plant presence, even under consistent experimental conditions.

### 3.3 Ryegrass growth parameters

**Figure 3:**
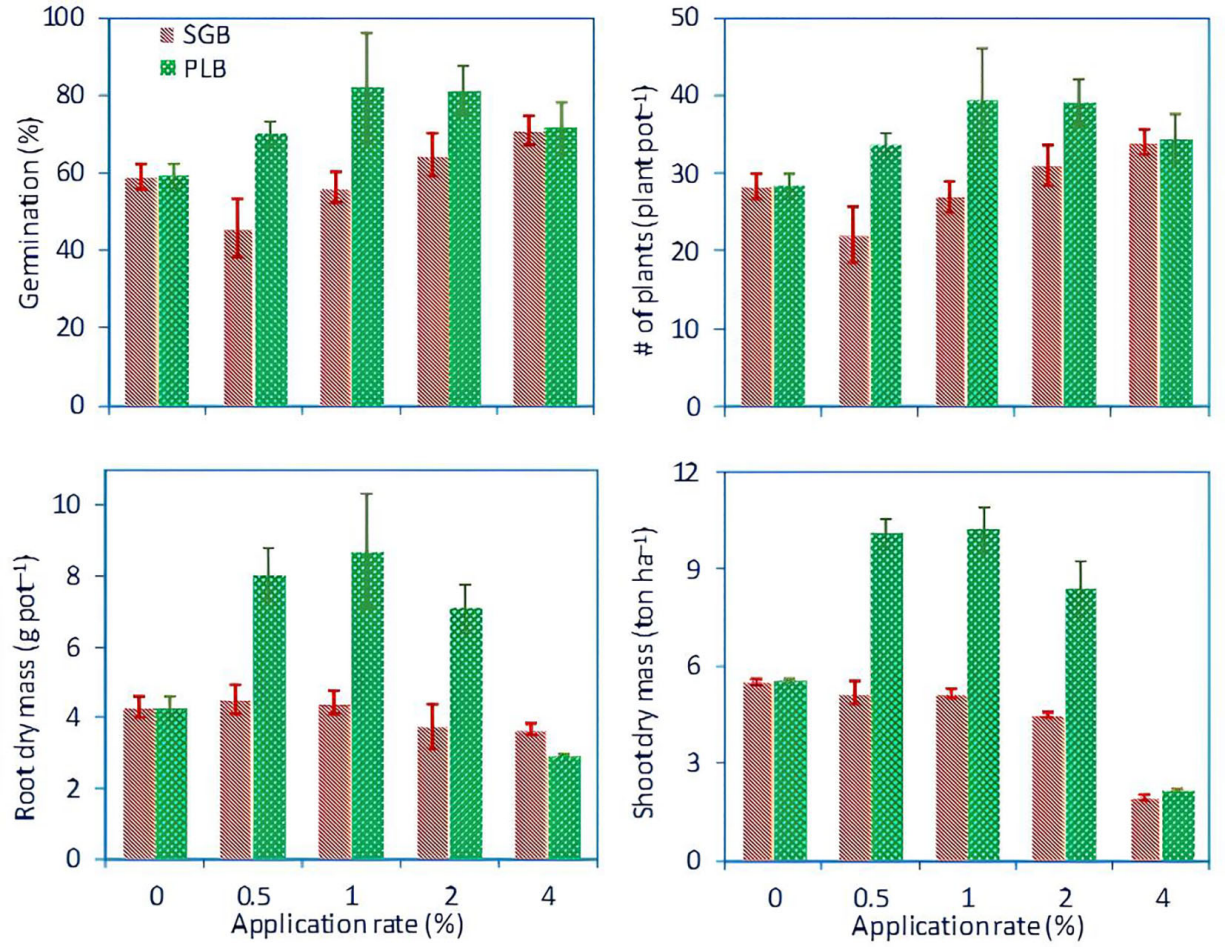
Average germination percentage, plant count per pot, root dry mass (g pot^-1^), and shoot dry mass (yield, ton ha^-1^) across different biochar treatment rates for ryegrass. Error bars represent standard deviations of the mean (*n*=3). SGB: biochar derived from switchgrass. PLB: biochar derived from poultry litter. This figure is an adaptation of the data presented in the work of Antonangelo and Zhang (2019), with the inclusion of plant population information.

The response of ryegrass to different rates of biochar, with measurements including mean percentage (%) of germination, number of plants per pot, root dry mass (grams pot^-1^), and shoot dry mass (yield, ton ha^-1^) are illustrated in **Figure 3**. High germination rates, plants population, and biomass production were evident in treatments with 0.5%, 1%, and 2% PLB, while other treatments exhibited poor germination and low biomass. This outcome aligns with findings by Antonangelo and Zhang (2019), who highlighted the role of high salinity (electrical conductivity, EC > 4 dS m^-1^) in impeding plant growth. Environmental stresses, such as elevated osmotic pressure and metallic ion accumulation, disrupt electron transport processes, leading to reactive oxygen species (ROS) overproduction (Bidar et al., 2007). In turn, ROS induces oxidative damage to lipids, proteins, and nucleic acids, hindering plant development. Under saline conditions, significant reductions in emergence rate and the proportion of forage crops have also been observed elsewhere in the literature (Kumar et al., 2021; Ibrahim et al., 2020; Menezes et al., 2017; Batool et al., 2015).

Regarding shoot biomass, it is evident that the application of PLB positively influenced ryegrass growth and yield. Specifically, PLB application at concentrations of 0.5% and 1% resulted in a notable increase in ryegrass growth, approximately 46% higher compared to the control (**Figure 3**). However, a subsequent decrease of approximately 17% was observed at the 2% PLB application rate, with a dramatic reduction of 79% observed at the highest application rate of 4% (**Figure 3**). These findings may suggest a negative correlation with salinity stress (r = -0.84, p<.001), as evidenced by EC measurements falling below the established threshold of 4.0 dS m^-1^ for PLB rates applied at 0.5% (EC = 3.5 dS m^-1^) and 1% (EC = 3.9 dS m^-1^), as reported by Antonangelo and Zhang (2019). However, higher EC levels of 4.5 and 6.9 dS m^-1^ were observed for application rates of 2% and 4%, respectively (Antonangelo and Zhang, 2019), warranting further investigation.

The observation that PLB application at 0.5% to 1% concentrations yielded greater root and shoot biomass suggests that seed food/energy reserves may not have been fully utilized at these biochar rates, thereby enabling biochar to conserve reserves and enhance overall yield. Conversely, at higher PLB concentrations (2 to 4%), it is plausible that seed reserves were depleted, as evidenced by comparable germination rates (while yield parameters for ryegrass were drastically different). In such instances, physiological reserves were likely insufficient to support subsequent plant growth at the highest PLB application rates. Past studies have demonstrated that while the total germination of perennial ryegrass remains unaffected by high salinity levels, the yields of mature plant growth experience a notable reduction of 50% (Maas and Hoffman, 1977). Subsequent research by Dudeck and Peacock (1985) further elucidated that perennial ryegrass exhibits greater salt tolerance during germination compared to later stages of growth. Therefore, careful consideration of biochar dosage is paramount in managing land-degraded and contaminated soils to optimize crop growth while adhering to environmental regulations regarding metal immobilization and mitigating risks associated with nutrient accumulation and salinity.

The application of SMLR analysis elucidated the intricate relationship between soil chemical attributes and ryegrass growth parameters, as presented in **Table 2**. Notably, the SMLR models demonstrated remarkable similarity for plant germination and population across both biochars, indicative of the anticipated positive correlation between these growth indicators. However, for PLB (**Table 2**), elevated soil Mg levels emerged as a potential impediment to plant germination, attributed to pH elevation, nutrient imbalance, toxicity, physical barriers such as compaction, and osmotic stress. This underscores the imperative of balanced soil management strategies for successful germination outcomes. Similarly, evaluating SGB (**Table 2**) reveals a comparable scenario with soil Ca levels. Conversely, both Boron (B) and Organic Carbon (OC) exhibited a positive influence on plant germination when SGB was employed. Boron’s pivotal role in physiological processes, encompassing cell elongation and membrane integrity, underscores its significance in promoting seed germination and subsequent seedling vigor. Moreover, OC’s beneficial effects on soil structure, water retention, and nutrient availability foster conducive conditions for seed germination and early seedling growth. Therefore, it is hypothesized that SGB applications facilitated optimal levels of B and OC in the soil, thereby enhancing seedling establishment. Regarding plant biomass yield (roots and shoots), irrespective of biochar type, adverse effects were observed concerning pH and P levels (**Table 2**). Extreme pH values disrupt nutrient availability, with alkalinity particularly impeding nutrient uptake, while excess phosphorus disrupts nutrient equilibrium, posing ecological risks. All equations derived from the SML models had regression coefficients that were significant at least at the p<.05 level. Subsequently, the intricate relationship between soil-available nutrients and their uptake by plants is discussed in the subsequent section.

**Table 2:** Stepwise multiple linear regression for soil chemical attributes affecting ryegrass growth parameters.

Ryegrass	———————— SGB ————————		
	Equation	R^2^	*p-value*
Germination	67.3 + (5.71×OC) – (9.31×Ca) + (5.62×B)	0.75	0.001
# of plants	32.3 + (2.74×OC) – (4.47×Ca) + (2.70×B)	0.75	0.001
Roots yield	8.17 – (0.40×pH) – (1.08×P)	0.46	0.02
Shoots yield	8.17 – (1.12×OC)	0.92	<.0001
———————— PLB ————————			
	Equation	R^2^	*p-value*
Germination	120 – (18.4×Mg)	0.49	0.004
# of plants	57.6 – (8.87×Mg)	0.49	0.004
Roots yield	-7.99 + (0.29×K) + (1.59×S)	0.90	<.0001
Shoots yield	52.4 – (5.39×pH) – (3.41×P) +(0.52×K) – (5.13×Mg)	0.96	<.0001

SGB, switchgrass-derived biochar; PLB, poultry litter-derived biochar.

### 3.4 Nutrients availability and plants uptake

**Figure 4:**
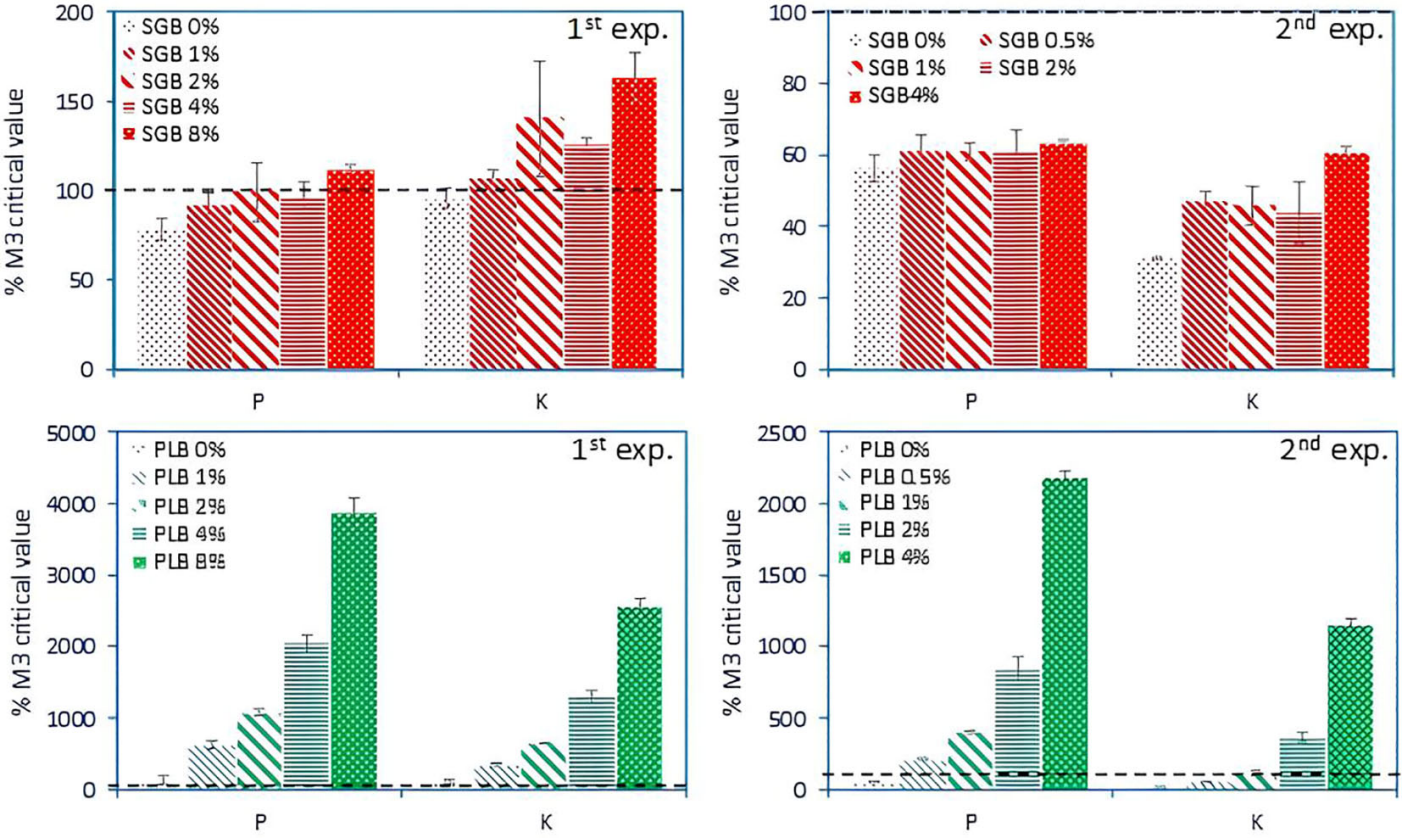
Changes in soil available P and K as a function of increased biochar application rates after 10 and 15 weeks of incubation, respectively for the Experiments 1 and 2. Data shown are as a percentage of the critical concentrations of 32.5 and 125 mg kg^-1^ for Mehlich-3 (M3) extractable P and K, respectively, for 100% sufficiency of ryegrass production (Zhang et al., 2017). Bars indicate the standard deviation of the mean (*n*=3). SGB: switchgrass-derived biochar. PLB: poultry litter-derived biochar. The black dashed line indicates the 100% sufficiency level for maximum/optimum ryegrass yield (and several other crops). Note the different scales among graphs.

The increase of extractable concentrations of P and K (also considered as plant available) in response to biochar application was compared to soil critical values published by Zhang et al. (2017) for Oklahoma (**Figure 4**) (absolute values of P and K concentrations can be seen in **Supplementary Tables S1**-**S4**). Despite the application of 4% of SGB during Experiment 2, soil extractable P and K concentrations remained below the critical thresholds of 32.5 and 125 mg kg^-1^ for P and K, respectively (**Figure 4**) for ryegrass cultivation. Notably, in the absence of plant cultivation (Experiment 1), a lower application rate of 2% of SGB was adequate to achieve 100% sufficiency levels for both nutrients (**Figure 4**), highlighting the inherent nutrient deficiency of the soils (as observed with the *control*―0% treatment) and the capacity of ryegrass to efficiently utilize these nutrients upon biochar application. The application of 2 to 4% SGB resulted in a near-complete sufficiency level of P in Experiment 1, leading to enhanced ryegrass uptake in the subsequent trial and consequently a reduction in soil extractable P (**Figure 4**). However, while 2-4% SGB application initially exceeded 100% sufficiency for K during Experiment 1, this level decreased notably during Experiment 2, indicating a phenomenon of K luxury uptake, particularly by perennial ryegrass (Ebdon et al., 2013).

In contrast, the application of PLB, characterized by its higher ash content and elemental concentrations (**Table 1**), resulted in significantly higher concentrations of P and K compared to SGB (**Figure 4**). Notably, even at a lower application rate of 1% PLB, soil extractable P and K concentrations surpassed 100% sufficiency levels for ryegrass cultivation across both experiments (**Figure 4**). This observation elucidates the superior performance of PLB in elevating P and K levels, contributing to improved plant parameters, particularly notable at the 0.5-1% PLB application rates during Experiment 2 (**Figure 3**), where P and K concentrations approached the adequate levels for maximizing ryegrass yield (**Figures 3**, **4**). As the application rate exceeded 1% of PLB, it fell into the ‘very high’ category (500%+ the critical level), indicating potential harm to the environment and/or plants, particularly concerning P (van Doorn et al., 2023). Furthermore, escalating PLB application rates lead to concurrent increases in soil EC, posing salinity damages for ryegrass cultivation, as previously mentioned. This illustrates that, when applied at a rate of 1% PLB, most heavy metals become immobilized (Antonangelo and Zhang, 2019), leading to the optimal growth of ryegrass and the maintenance of nutrient levels at approximately 100% sufficiency. However, exceeding the 1% application rate may result in diminished immobilization of metals, albeit to a lesser degree (Antonangelo and Zhang, 2019), and could potentially exacerbate another environmental concern due to the accumulation of P.

These findings reinforce the work of Ginebra et al. (2022), who reported that applying pig manure biochar and poultry litter-derived carbonaceous materials at a rate of 1% can effectively improve key soil properties in forage systems. Specifically, these amendments were found to raise soil pH and boost available P levels, which are critical for crop growth. The benefits were especially pronounced in Andisols with an initial pH of approximately 5.9, where such amendments not only enhanced soil fertility but also contributed to greater crop productivity. This supports the idea that organic amendments tailored to acidic soils can play an important role in sustainable soil management by fostering improved nutrient availability and soil health.

**Figure 5:**
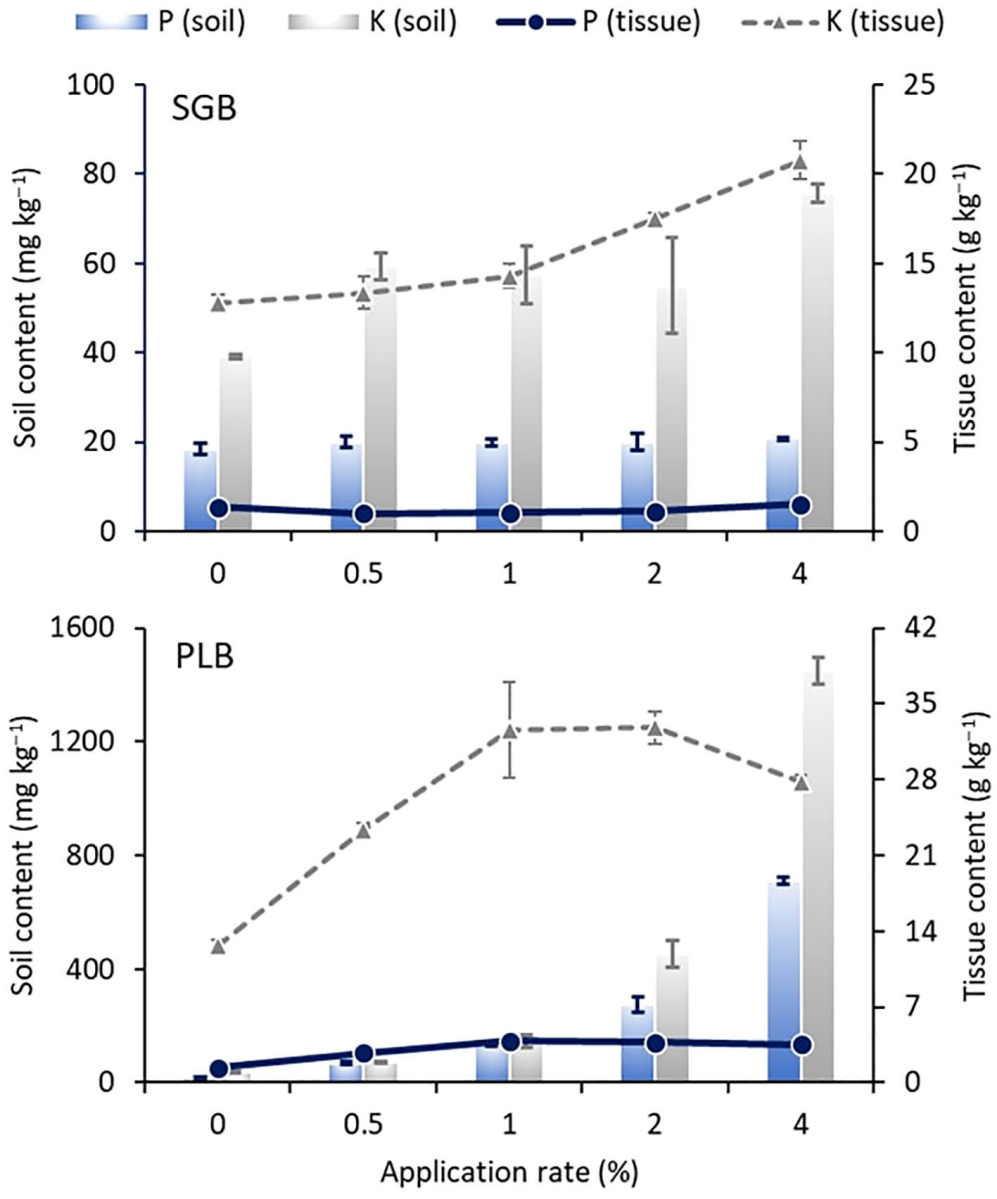
Dynamics of soil available phosphorus (P) and potassium (K) content (columns) and plant uptake of P and K (lines) in response to incremental biochar application following a 15-week incubation period under ryegrass cultivation. Error bars represent standard deviation of the mean (*n*=3). Biochar types include SGB (switchgrass-derived biochar) and PLB (poultry litter-derived biochar). Please note the varying scales depicted in the graphs.

Across experiments, variations in the extent to which biochar augmented extractable nutrient concentrations, relative to critical levels, were observed between biochars and experimental setups (**Figure 4**). Notably, the concentrations of extractable P and K exhibited linear increases in the first trial (Experiment 1), specifically when using SGB, respectively yielding the equations *y = 3.6x + 85 (R^2^ = 0.59, p<.01)* and *y = 7.5x + 104 (R^2^ = 0.61, p<.01)*. This trend can be attributed to the fact that 8% of SGB surpassed the 100% sufficiency threshold for P in Experiment 1, while all other rates remained at or below this threshold. Subsequently, in Experiment 2, only up to 4% of SGB was applied. Consequently, similar levels of plant P uptake were observed (**Figure 5**) and no linear increment was observed. Regarding K, where luxury uptake occurs and soil-available K exceeds 100% sufficiency levels starting at a 2% SGB application rate (**Figure 4**), a slight increase in both soil-available levels and plant uptake was observed (**Figure 5**), and a linear model was successfully obtained: *y = 5.6x + 37.4 (R^2^ = 0.62, p<.001)*.

In the context of PLB, a consistent linear and exponential trend emerged across P and K for the 1^st^ and 2^nd^ experiments, respectively, with increasing biochar application rates leading to substantial gains. These trends are strongly supported by high R² values (0.99 for Experiment 1 and between 0.96 and 0.97 for Experiment 2), indicating a robust relationship between biochar application and nutrient availability. Notably, the 0.5–1% application rate was optimal for ryegrass performance (**Figure 3**), aligning with the threshold uptake of P and K (**Figures 4**, **5**), which correlates with soil levels that support maximum growth.

Regarding other essential nutrients such as Ca, Mg, S, Fe, B, and Cu, discernible variations in soil available contents were observed across applied rates of biochars. Notably, throughout the experiments, PLB consistently outperformed SGB in most cases, except for Cu in the second experiment (**Supplementary Tables S1**-**S4**). This trend was corroborated by aboveground plant uptake in Experiment 2, apart from an inverse pattern observed for S, Ca, and Mg (**Supplementary Tables S5**, **S6**). The observed decline in the uptake of these secondary macronutrients when PLB was applied, in contrast to SGB, could potentially be attributed to the excessive levels of P and K supplied by PLB, with their uptake possibly enhanced (and favored) by the overall higher N uptake with the application of this biochar (**Supplementary Tables S5**). This abundance might lead to the inhibition of secondary macronutrient availability due to chemical reactions resulting in precipitation and competitive interactions for plant uptake. This interpretation gains support from a subsequent simple correlation analysis (Pearson), which reveals highly significant and negative correlations between P and K with Ca, Mg, and S in both soil and plant roots and shoots (**Supplementary Figure S1**). Even when considering data encompassing all biochars (**Supplementary Figure S1**), the trends associated with increases in P and K levels can confidently be ascribed to PLB, as its levels in the soil significantly surpassed those observed when SGB was applied.

## 4 Conclusion

The study investigated the efficacy of SGB and PLB in improving soil pH and nutrient availability for ryegrass growth in a metal-contaminated soil. Two experiments were conducted: one focusing solely on biochars’ effects and another assessing combined biochar and ryegrass growth impacts. Results revealed that feedstock origin rather than pyrolysis temperature influenced biochar’s buffering capacity, with PLB exhibiting greater buffering capacity than SGB. The sustained increase in soil pH over 10 to 15 weeks indicated biochar’s long-term effectiveness in pH management, crucial for maintaining optimal nutrient availability and microbial activity in agricultural systems.

Application of PLB significantly enhanced ryegrass productivity across various parameters, with the greatest improvement observed at a 1% application rate. Excessive biochar application (>2-4%) had adverse effects on plant growth, emphasizing the need for careful dosage control. The 1% PLB application rate promoted 100% sufficiency levels of P and K for ryegrass growth while yielding optimal root and shoot biomass. In conclusion, PLB biochar shows promise as a soil amendment to boost ryegrass productivity, but careful dosage management is essential to maximize benefits and avoid negative impacts on plant growth. Future research should investigate specific mechanisms of biochar interaction with soil microbial communities and nutrient cycling processes to optimize application rates and methods that enhance crop productivity and soil health in diverse agricultural systems.

## Data Availability

The raw data supporting the conclusions of this article will be made available by the authors, without undue reservation.
