# Supplementary material for: Fertilizer potential of biochar and ryegrass productivity in metal-contaminated soil
Source: Front Plant Sci. 2025 Jan 9;15:1475939. doi: 10.3389/fpls.2024.1475939 (PMC11754300; doi:10.3389/fpls.2024.1475939)
Supplement: Supplementary file 1 [file DataSheet1.docx]

***Supplementary Table S1.*** ANOVA for the soil chemical attributes, macro- and micronutrients as a function of increased biochar application rates (Experiment 1).

| Rate | --------- pH --------- | | | --------- OC, % --------- | | | --------- P, mg kg^‒1^ --------- | | | --------- S, mg kg^‒1^ --------- | | |
| --- | --- | --- | --- | --- | --- | --- | --- | --- | --- | --- | --- | --- |
| % | SGB | PLB | Avg | SGB | PLB | Avg | SGB | PLB | Avg | SGB | PLB | Avg |
| 0 | 7.2 bA | 7.2 cA | 7.2 c | 2.1 eA | 2.1 dA | 2.1 e | 25.5 bA | 25.5 eA | 25.5 e | 38.6 aA | 38.6 dA | 38.6 d |
| 1 | 7.2 bA | 6.8 dB | 7.0 d | 2.9 dA | 2.3 dB | 2.6 d | 30.2 abB | 202 dA | 116 d | 36.4 abB | 113 cA | 74.7 c |
| 2 | 7.2 bA | 7.1 cA | 7.2 c | 4.0 cA | 2.7 cB | 3.3 c | 32.3 abB | 349 cA | 191 c | 36.8 abB | 137 cA | 87.1 c |
| 4 | 7.3 abA | 7.4 bA | 7.4 b | 5.4 bA | 3.2 bB | 4.3 b | 31.7 abB | 666 bA | 349 b | 29.5 bcB | 226 bA | 128 b |
| 8 | 7.5 aB | 8.0 aA | 7.7 a | 7.9 aA | 4.0 aB | 6.0 a | 36.5 aB | 1259 aA | 648 a | 25.9 cB | 586 aA | 306 a |
| Avg | 7.3 A | 7.3 A |  | 4.5 A | 2.9 B |  | 31.2 B | 500 A |  | 33.4 B | 220 A |  |
| *Effect test* | *p-value ---------------* | | | | | | | | | | | |
| *Biochar* | 0.1644 | | | <.0001 | | | <.0001 | | | <.0001 | | |
| *Rate* | <.0001 | | | <.0001 | | | <.0001 | | | <.0001 | | |
| *Biochar×Rate* | <.0001 | | | <.0001 | | | <.0001 | | | <.0001 | | |
| Rate |  | --------- Fe, mg kg^‒1^ --------- | | | --------- B, mg kg^‒1^ --------- | | | --------- Cu, mg kg^‒1^ --------- | | |  |  |
| % |  | SGB | PLB | Avg | SGB | PLB | Avg | SGB | PLB | Avg |  |  |
| 0 |  | 22.5 aA | 22.5 aA | 22.5 a | 0.15 aA | 0.15 dA | 0.15 d | 1.1 aA | 1.1 cA | 1.1 c |  |  |
| 1 |  | 18.7 aA | 20.9 abA | 19.8 ab | 0.11 aB | 0.38 cA | 0.25 c | 0.9 aB | 1.3 cA | 1.1 c |  |  |
| 2 |  | 18.0 aA | 17.6 bcA | 17.8 bc | 0.12 aB | 0.52 cA | 0.32 c | 0.8 aB | 1.4 cA | 1.1 c |  |  |
| 4 |  | 18.8 aA | 15.3 cdA | 17.1 bc | 0.12aB | 0.88 bA | 0.50 b | 0.9 aB | 1.7 bA | 1.3 b |  |  |
| 8 |  | 17.9 aA | 11.8 dB | 14.9 c | 0.13 aB | 1.53 aA | 0.83 a | 0.9 aB | 2.4 aA | 1.6 a |  |  |
| Avg |  | 19.2 A | 17.6 B |  | 0.13 B | 0.69 A |  | 0.9 B | 1.6 A |  |  |  |
| *Effect test* | *p-value ---------------* | | | | | | | | | | | |
| *Biochar* |  | 0.026 | | | <.0001 | | | <.0001 | | |  |  |
| *Rate* |  | <.0001 | | | <.0001 | | | <.0001 | | |  |  |
| *Biochar×Rate* |  | 0.007 | | | <.0001 | | | <.0001 | | |  |  |

Different lowercase letters in columns and uppercase letters in rows are significantly different from the Tukey test at α=.05. OC: organic carbon. SGB: switchgrass-derived biochar. PLB: poultry litter-derived biochar.

***Supplementary Table S2.*** ANOVA for the soil chemical attributes, macro- and micronutrients as a function increased biochar application rates (Experiment 2).

| Rate | --------- pH --------- | | | --------- OC, % --------- | | | --------- P, mg kg^‒1^ --------- | | | --------- S, mg kg^‒1^ --------- | | | |
| --- | --- | --- | --- | --- | --- | --- | --- | --- | --- | --- | --- | --- | --- |
| % | SGB | PLB | Avg | SGB | PLB | Avg | SGB | PLB | Avg | SGB | PLB | Avg |  |
| 0 | 5.9 dA | 5.9 dA | 5.9 e | 2.1 dA | 2.1 cA | 2.1 e | 18.3 aA | 18.3 eA | 18.3 e | 491 aA | 491 aA | 491 a |  |
| 0.5 | 6.7 cA | 6.0 dB | 6.4 d | 2.5 dA | 2.3 bcA | 2.4 d | 20.0 aB | 69 dA | 44.5 d | 115 bB | 422 abA | 269 b |  |
| 1 | 6.9 bcA | 6.3 cB | 6.6 c | 3.0 cA | 2.5 bB | 2.7 c | 19.8 aB | 132 cA | 75.8 c | 85.8 bB | 344 bA | 215 c |  |
| 2 | 7.0 bA | 6.8 bB | 6.9 b | 3.8 bA | 2.9 aB | 3.3 b | 20.0 aB | 275 bA | 147 b | 64.0 bcB | 186 cA | 125 d |  |
| 4 | 7.5 aA | 7.6 aB | 7.5 a | 5.3 aA | 3.0 aB | 4.2 a | 20.7 aB | 711 aA | 366 a | 14.5 cB | 188 cA | 101 d |  |
| Avg | 6.8 A | 6.5 B |  | 3.3 A | 2.6 B |  | 19.8 B | 241 A |  | 154 B | 326 A |  |  |
| *Effect test* | *p-value ---------------* | | | | | | | | | | | | |
| *Biochar* | <.0001 | | | <.0001 | | | <.0001 | | | <.0001 | | | |
| *Rate* | <.0001 | | | <.0001 | | | <.0001 | | | <.0001 | | | |
| *Biochar×Rate* | <.0001 | | | <.0001 | | | <.0001 | | | <.0001 | | | |
| Rate |  | --------- Fe, mg kg^‒1^ --------- | | | --------- B, mg kg^‒1^ --------- | | | --------- Cu, mg kg^‒1^ --------- | | |  |  |  |
| % |  | SGB | PLB | Avg | SGB | PLB | Avg | SGB | PLB | Avg |  |  |  |
| 0 |  | 29.8 aA | 29.8 aA | 29.8 a | 0.12 bcA | 0.12 eA | 0.12 e | 1.4 bA | 1.4 cA | 1.4 c |  |  |  |
| 0.5 |  | 29.5 aA | 33.6 aA | 31.6 a | 0.18 aB | 0.24 dA | 0.21 d | 1.7 abA | 1.7 bcA | 1.7 abc |  |  |  |
| 1 |  | 23.9 bA | 29 aA | 26.4 b | 0.15 abB | 0.37 cA | 0.26 c | 1.7 abA | 1.7 bcA | 1.7 bc |  |  |  |
| 2 |  | 21.3 bcA | 21.6 bA | 21.5 c | 0.12 bcB | 0.56 bA | 0.34 b | 2.3 aA | 2.0 bA | 2.2 a |  |  |  |
| 4 |  | 18.4 cA | 17.3 bA | 17.9 d | 0.08 cB | 1.0 aA | 0.54 a | 1.5 abB | 2.4 aA | 2.0 ab |  |  |  |
| Avg |  | 24.6 B | 26.2 A |  | 0.13 B | 0.46 A |  | 1.73 A | 1.87 A |  |  |  |  |
| *Effect test* | *p-value ---------------* | | | | | | | | | | | | |
| *Biochar* |  | 0.019 | | | <.0001 | | | 0.1463 | | |  |  |  |
| *Rate* |  | <.0001 | | | <.0001 | | | 0.0005 | | |  |  |  |
| *Biochar×Rate* |  | 0.030 | | | <.0001 | | | 0.0086 | | |  |  |  |

Different lowercase letters in columns and uppercase letters in rows are significantly different from the Tukey test at α=.05. OC: organic carbon. SGB: switchgrass-derived biochar. PLB: poultry litter-derived biochar.

***Supplementary Table S3.*** ANOVA for the macronutrients and cation exchange capacity as a function of increased biochar application rates (Experiment 1).

| Rate | --------- K, mg kg^‒1^ --------- | | | --------- Ca, mg kg^‒1^ --------- | | |
| --- | --- | --- | --- | --- | --- | --- |
| % | SGB | PLB | Avg | SGB | PLB | Avg |
| 0 | 120 cA | 120 eA | 120 e | 2035 aA | 2035 dA | 2035 b |
| 1 | 135 bcB | 441 dA | 288 d | 2006 aB | 2139 cdA | 2072 b |
| 2 | 158 abcB | 814 cA | 495 c | 1948 aB | 2248 bcA | 2098 b |
| 4 | 176 abB | 1626 bA | 892 b | 1781 bB | 2422 bA | 2102 b |
| 8 | 205 aB | 3215 aA | 1710 a | 1626 cB | 2799 aA | 2212 a |
| Avg | 159 B | 1243 A |  | 1879 B | 2328 A |  |
| *Effect test* | *p-value ---------------* | | | | | |
| *Biochar* | <.0001 | | | <.0001 | | |
| *Rate* | <.0001 | | | 0.0002 | | |
| *Biochar×Rate* | <.0001 | | | <.0001 | | |
| Rate | --------- Mg, mg kg^‒1^ --------- | | | --------- CEC, cmol kg^‒1^ --------- | | |
| % | SGB | PLB | Avg | SGB | PLB | Avg |
| 0 | 160 aA | 160 eA | 160 e | 11.8 aA | 11.8 eA | 11.8 e |
| 1 | 156 aB | 259 dA | 208 d | 11.7 aB | 14.0 dA | 12.8 d |
| 2 | 166 aB | 336 cA | 251 c | 11.6 aB | 16.1 cA | 13.9 c |
| 4 | 152 aB | 507 bA | 330 b | 10.6 bB | 20.5 bA | 15.5 b |
| 8 | 156 aB | 831 aA | 494 a | 10.0 bB | 29.2 aA | 19.6 a |
| Avg | 158 B | 419 A |  | 11.1 B | 18.3 A |  |
| *Effect test* | *p-value ---------------* | | | | | |
| *Biochar* | <.0001 | | | <.0001 | | |
| *Rate* | <.0001 | | | <.0001 | | |
| *Biochar×Rate* | <.0001 | | | <.0001 | | |

Different lowercase letters in columns and uppercase letters in rows are significantly different from the Tukey test at α=.05. CEC: cation exchange capacity (Ca+Mg+K). SGB: switchgrass-derived biochar. PLB: poultry litter-derived biochar.

***Supplementary Table S4.*** ANOVA for the macronutrients and cation exchange capacity as a function of increased biochar application rates (Experiment 2).

| Rate | --------- K, mg kg^‒1^ --------- | | | --------- Ca, mg kg^‒1^ --------- | | |
| --- | --- | --- | --- | --- | --- | --- |
| % | SGB | PLB | Avg | SGB | PLB | Avg |
| 0 | 39 cA | 39.0 dA | 39.0 e | 2696 aA | 2696 aA | 2696 a |
| 0.5 | 59.3 bB | 70.3 cdA | 64.8 d | 2368 abA | 2306 bA | 2337 b |
| 1 | 57.3 bB | 147 cA | 102 c | 2185 bA | 2200 bcA | 2192 bc |
| 2 | 55 bB | 454 bA | 254 b | 1942 bcA | 2049 cA | 1996 c |
| 4 | 75.8 aB | 1451 aA | 763 a | 1654 cB | 2540 aA | 2097 c |
| Avg | 57.3 B | 432 A |  | 2169 B | 2358 A |  |
| *Effect test* | *p-value ---------------* | | | | | |
| *Biochar* | <.0001 | | | 0.0009 | | |
| *Rate* | <.0001 | | | <.0001 | | |
| *Biochar×Rate* | <.0001 | | | <.0001 | | |
| Rate | --------- Mg, mg kg^‒1^ --------- | | | --------- CEC, cmol kg^‒1^ --------- | | |
| % | SGB | PLB | Avg | SGB | PLB | Avg |
| 0 | 216 aA | 216 dA | 216 bc | 15.4 aA | 15.4 bA | 15.4 a |
| 0.5 | 172 bB | 230 dA | 201 c | 13.4 abA | 13.6 cA | 13.5 b |
| 1 | 149 bcB | 275 cA | 212 bc | 12.3 bA | 13.7 cA | 13.0 b |
| 2 | 141 cB | 318 bA | 229 b | 11.0 bcB | 14.1 bcA | 12.5 b |
| 4 | 126 cB | 517 aA | 321 a | 9.52 cB | 20.7 aA | 15.1 a |
| Avg | 161 B | 311 A |  | 12.3 B | 15.5 A |  |
| *Effect test* | *p-value ---------------* | | | | | |
| *Biochar* | <.0001 | | | 0.0009 | | |
| *Rate* | <.0001 | | | <.0001 | | |
| *Biochar×Rate* | <.0001 | | | <.0001 | | |

Different lowercase letters in columns and uppercase letters in rows are significantly different from the Tukey test at α=.05. CEC: cation exchange capacity (Ca+Mg+K). SGB: switchgrass-derived biochar. PLB: poultry litter-derived biochar.

***Supplementary Table S5.*** ANOVA for the ryegrass macronutrients status aboveground as a function of increased biochar application rates (Experiment 2).

| Rate | --------- N, g kg^‒1^ --------- | | | --------- P, g kg^‒1^ --------- | | | --------- K, g kg^‒1^ --------- | | |
| --- | --- | --- | --- | --- | --- | --- | --- | --- | --- |
| % | SGB | PLB | Avg | SGB | PLB | Avg | SGB | PLB | Avg |
| 0 | 40.0 aA | 40.0 aA | 40.0 a | 1.37 abA | 1.37 cA | 1.37 c | 12.7 cA | 12.7 cA | 12.7 a |
| 0.5 | 21.9 bB | 28.4 bA | 25.2 b | 0.99 cB | 2.78 bA | 1.88 b | 13.4 cB | 23.4 bA | 18.4 b |
| 1 | 19.2 bcB | 26.2 bcA | 22.7 b | 1.03 cB | 3.88 aA | 2.46 a | 14.3 cB | 32.6 aA | 23.4 a |
| 2 | 16.1 cB | 21.5 cA | 18.8 c | 1.13 bcB | 3.71 aA | 2.42 a | 17.5 bB | 32.8 aA | 25.2 a |
| 4 | 9.80 dB | 13.2 dA | 11.5 d | 1.51 aB | 3.56 aA | 2.54 a | 20.8 aB | 27.8 abA | 24.3 a |
| Avg | 21.4 B | 25.9 A |  | 1.21 B | 3.06 A |  | 15.7 B | 25.9 A |  |
| *Effect test* | *p-value ---------------* | | | | | | | | |
| *Biochar* | 0.019 | | | <.0001 | | | <.0001 | | |
| *Rate* | <.0001 | | | <.0001 | | | <.0001 | | |
| *Biochar×Rate* | 0.005 | | | <.0001 | | | <.0001 | | |
| Rate | --------- S, g kg^‒1^ --------- | | | --------- Ca, g kg^‒1^ --------- | | | --------- Mg, g kg^‒1^ --------- | | |
| % | SGB | PLB | Avg | SGB | PLB | Avg | SGB | PLB | Avg |
| 0 | 5.54 aA | 5.54 aA | 5.54 a | 11.0 aA | 11.0 aA | 11.0 a | 3.30 aA | 3.30 aA | 3.30 a |
| 0.5 | 3.69 bB | 5.46 abA | 4.57 b | 8.94 aA | 6.59 bB | 7.76 b | 2.89 aA | 2.55 bB | 2.72 b |
| 1 | 3.88 bA | 4.62 bA | 4.25 b | 8.73 aA | 5.44 bcB | 7.09 b | 3.07 aA | 2.33 bB | 2.70 b |
| 2 | 3.69 bA | 3.23 cA | 3.46 b | 7.92 aA | 4.92 cB | 6.42 b | 2.82 aA | 2.38 bB | 2.60 b |
| 4 | 2.81 cA | 1.89 dB | 2.35 d | 9.90 aA | 3.33 dB | 6.61 b | 3.42 aA | 2.24 bA | 2.83 ab |
| Avg | 3.92 B | 4.15 A |  | 9.29 A | 6.25 B |  | 3.10 A | 2.56 B |  |
| *Effect test* | *p-value ---------------* | | | | | | | | |
| *Biochar* | 0.043 | | | <.0001 | | | <.0001 | | |
| *Rate* | <.0001 | | | <.0001 | | | 0.004 | | |
| *Biochar×Rate* | <.0001 | | | <.0001 | | | 0.022 | | |

Different lowercase letters in columns and uppercase letters in rows are significantly different from the Tukey test at α=.05.

***Supplementary Table S6.*** ANOVA for the ryegrass micronutrients status aboveground as a function of increased biochar application rates (Experiment 2).

| Rate | --------- Fe, mg kg^‒1^ --------- | | | --------- B, mg kg^‒1^ --------- | | | ---------Cu, mg kg^‒1^ --------- | | |
| --- | --- | --- | --- | --- | --- | --- | --- | --- | --- |
| % | SGB | PLB | Avg | SGB | PLB | Avg | SGB | PLB | Avg |
| 0 | 622 aA | 622 aA | 622 A | 14.4 aA | 14.4 aA | 14.4 a | 3.028 aA | 3028 aA | 3028 A |
| 0.5 | 159 aA | 90.5 aB | 125 A | 9.0 bB | 15.2 aA | 12.1 a | 22.1 aA | 15.4 aA | 18.7 b |
| 1 | 162 aA | 115 aA | 139 A | 9.1 abB | 16.1 aA | 12.6 a | 28.4 aA | 16.8 aA | 22.6 b |
| 2 | 101 aA | 86.1 aA | 93.8 A | 8.9 bB | 17.1 aA | 13.0 a | 16.4 aA | 15.2 aA | 15.8 b |
| 4 | 1585 aA | 90.8 aA | 838 A | 11.6 abA | 14.9 aA | 13.3 a | 74.2 aA | 40.6 aA | 57.4 b |
| Avg | 526 A | 201 B |  | 10.6 B | 15.5 A |  | 634 A | 623 A |  |
| *Effect test* | *p-value ---------------* | | | | | | | | |
| *Biochar* | 0.327 | | | <.0001 | | | 0.327 | | |
| *Rate* | 0.482 | | | 0.4903 | | | 0.001 | | |
| *Biochar×Rate* | 0.529 | | | 0.035 | | | 0.529 | | |

Different lowercase letters in columns and uppercase letters in rows are significantly different from the Tukey test at α=.05.

***Supplementary Figure S1.*** Pearson correlation between several variables analyzed in Experiment 2.

Green: p<.0001. Yellow: p<.001. Blue: p<.01. Grey: p<.05. Red: non-significant (p>.05). Results comprise the whole dataset of measurements (*n*=30.
